# Country‐Level Gender Equality and Adolescents’ Contraceptive Use in Europe, Canada and Israel: Findings from 33 Countries

**DOI:** 10.1363/psrh.12090

**Published:** 2019-02-28

**Authors:** Margaretha de Looze, Aubrey S. Madkour, Tim Huijts, Nathalie Moreau, Candace Currie

**Affiliations:** ^1^ Assistant professor, Department of Inter disciplinary Social Science, Faculty of Social and Behavioural Sciences Utrecht University Utrecht the Netherlands; ^2^ Associate professor, Department of Global Community Health and Behavioral Sciences Tulane University School of Public Health and Tropical Medicine New Orleans; ^3^ Researcher, Research Centre for Education and the Labour Market, School of Business and Economics Maastricht University Maastricht the Netherlands; ^4^ Researcher, Service d'Information Promotion Education Santé, School of Public Health Université Libre de Bruxelles Brussels Belgium; ^5^ Professor, Child and Adolescent Health Research Unit, WHO Collaborating Centre for International Child and Adolescent Health Policy, School of Medicine University of St. Andrews St. Andrews Scotland

## Abstract

**CONTEXT:**

Although an association between gender equality and contraceptive use has been confirmed among adult samples, few studies have explored this relationship among adolescents. An examination of whether adolescents’ contraceptive use is more prevalent in countries with higher levels of gender equality is needed to fill this gap.

**METHODS:**

Nationally representative data from 33 countries that participated in the 2013–2014 Health Behaviour in School‐Aged Children study and country‐level measures of gender equality—using the 2014 Global Gender Gap Index—were analyzed. Multilevel multinomial logistic regression analyses were employed to assess associations between gender equality and contraceptive use (condom only, pill only and dual methods) at last intercourse as reported by 4,071 females and 4,110 males aged 14–16.

**RESULTS:**

Increasing gender equality was positively associated with contraceptive use among both males and females. For every 0.1‐point increase on the equality scale, the likelihood of condom use at last intercourse rose (odds ratio, 2.1 for females), as did the likelihood of pill use (6.5 and 9.6, respectively, for males and females) and dual method use (2.1 and 5.6, respectively). Associations with pill use and dual use remained significant after national wealth and income inequality were controlled for. Overall, associations were stronger for females than for males.

**CONCLUSIONS:**

More research is needed to identify potential causal pathways and mechanisms through which gender equality and adolescents’ contraceptive use may influence one another.

Encouraging young people to practice safer sex is a major public health challenge^1^ that has been met with varying degrees of success worldwide. In Europe and North America, considerable cross‐national variation exists in contraceptive use among sexually active adolescents, and this variation has been consistent over time.^2^, ^3^, ^4^ In 2014, an international study using nationally representative samples showed that condom use at last sexual intercourse among sexually active 14–16‐year‐olds varied from 26% in Poland to 81% in Switzerland; pill use among this age‐group varied from 8% in the Republic of Moldova to 66% in Germany.^4^ Identifying the variables associated with adolescents’ contraceptive use may be useful for programs and interventions seeking to promote consistent contraceptive use in this population.

Although a number of studies have speculated that macro‐level gender equality may at least partly explain the cross‐national variation in adolescents’ contraceptive use,^5^, ^6^ studies testing this hypothesis are scarce. The main goal of the present study was to examine whether macro‐level gender equality is associated with contraceptive use among sexually experienced adolescents in Europe, Canada and Israel. Given the assumption that gender equality is linked to the cultural acceptability of contraceptive use and women's ability to actively participate in decision making regarding contraception, we hypothesized that contraceptive use would be more prevalent in countries with higher levels of gender equality than in countries with lower levels.

## Background

A full understanding of the patterns of contraceptive use among adolescents requires an ecological approach, involving an examination of individual and contextual variables, at different levels, and their interaction.^7^ Yet, the vast majority of studies on this topic focus only on individual‐level characteristics, such as a disrupted family structure,^8^, ^9^ the quality of parent‐child relationships and sexual communication,^10^, ^11^, ^12^ adolescent substance use,^13^ school attachment^11^ and socioeconomic status.^14^, ^15^ To our knowledge, only two studies have examined links between macro‐level variables and adolescents’ sexual activity in Europe and North America. The first showed that young women—but not young men—are less likely to be sexually experienced in countries with more conservative cultural norms regarding sexuality than in those with more liberal norms.^6^ The second demonstrated that country‐level indicators, including the Human Development Index, national wealth and income inequality, the predominant national religion and the national prevalence of HIV, are related to condom use among young Europeans.^16^ However, the latter study controlled for very few variables at the individual level and thus may have overestimated associations with macro‐level covariates. Other studies examining associations between contextual variables and adolescents’ contraceptive use have been conducted primarily in the United States^17^ or in developing countries.^18^ A comprehensive analysis of the potential associations between macro‐level and micro‐level characteristics and adolescents’ contraceptive use in Europe and North America is thus lacking.

Gender equality—here defined as the extent to which women and men have an equal share of paid work, money and decision‐making power in society—varies considerably across European and North American countries; Scandinavian countries typically have the highest rates of gender equality and eastern European countries the lowest.^19^ Among adults, macro‐level gender equality is associated with higher rates of contraceptive use, including condom and pill use.^20^ Gender equality may be linked to contraceptive use among adults through its associations with more equal distribution of resources (including power) within heterosexual romantic relationships,^20^ better communication between sexual partners^21^ and more egalitarian gender norms in society.^22^, ^23^ In line with classic resource theories, the partner with the greater resources (e.g., higher education or income) has greater influence in a couple's method choice.^24^ As women's resources rise relative to those of men, their engagement in the contraceptive decision‐making process increases. Given that women are generally more concerned about contraception than men because of their reproductive risk,^20^ contraceptive use may be higher in countries in which women have a more equal say in method choice. In addition, more egalitarian gender norms further support women in taking a more active role in contraceptive decision making.^22^, ^23^ Moreover, these norms stimulate men to define the shared decision making as part of their role as a responsible man and as a way of taking care of their partner, rather than as a threat to their dominance and masculinity.^25^

Although the link between gender equality and contraceptive use among adults has been confirmed, much less is known about how gender equality relates to contraceptive use among adolescents. Yet, there are reasons to assume that gender equality may be associated with cross‐national variability in contraceptive use among youth in particular. First, adolescents tend to be vulnerable to the influence of gender norms, as they are at a life stage in which consciousness of the need to “fit in” is highly salient and gender identities are actively being formed (or reformed).^26^ Deviating from the norms associated with one's biological sex can be met with social exclusion and censure from peers (e.g., a young woman may get a bad reputation if she carries condoms with her).^27^ More egalitarian gender norms may increase young women's confidence—even more than adult women's—to play a more active role in the contraceptive decision‐making process. Indeed, a review of more than 250 empirical studies concluded that greater female power in a relationship and greater acceptance of nontraditional gender roles for women were associated with increased contraceptive use among adolescents.^28^ Moreover, parental gender equality (e.g., maternal working time) has been related to young women's pill use in Sweden,^29^ and young men's support for gender‐equitable norms has been related to increased contraceptive use in Brazil^30^ and Canada.^31^

Second, countries with high levels of gender equality tend to invest more in comprehensive sex education^32^, ^33^ and youth‐friendly sexual and reproductive health services^34^ than countries with lower levels. As a result, young people in more gender‐equal countries are better equipped to make healthy and informed choices regarding sexuality. For example, owing to comprehensive sex education, adolescents in relatively gender‐equal countries may have more knowledge about the safest methods (i.e., dual methods), how to access contraceptives and how to use them. Youth‐friendly health services may increase contraceptive use through adequate financial reimbursement for the pill^34^, ^35^ and through the availability and accessibility of confidential services.^34^, ^36^, ^37^ All in all, it is expected that gender equality will be associated with young people's use of contraceptives.

Building on research showing that macro‐level gender equality is associated with more egalitarian gender norms,^22^, ^23^ more comprehensive sex education^32^, ^33^ and more youth‐friendly sexual and reproductive health services,^34^ the present study hypothesized that adolescents living in more gender‐equal countries were more likely to use contraceptives than their peers in less gender‐equal countries. Because pill use is under young women's control, and gender equality specifically refers to women's empowerment, we expected the association with gender equality to be especially strong for pill use. Finally, we expected dual method use to be more common in countries with higher levels of gender equality, because adolescents in these countries may be better informed about the safety of different methods.

## METHODS

### Data

Data for the present study come from the 2013–2014 Health Behaviour in School‐Aged Children (HBSC) study. This study was conducted in 42 nations, primarily in Europe, in collaboration with the World Health Organization's Regional Office for Europe. It was designed to examine the health and health behaviors of adolescents across country contexts.^4^

Data were collected through school‐based, anonymous surveys using a standard methodology.^38^ Each participating country or region used random sampling to select young people aged 11, 13 and 15, ensuring that the sample was representative of all in the age range. Fieldwork took place mainly between September 2013 and June 2014. The present analysis is limited to the 33 countries that participated in the 2013–2014 HBSC study, included the sexual behavior module for 15‐year‐olds and had a score on the Global Gender Gap Index in 2014.^39^ Response rates in these countries varied. At the school level, response rates ranged from 25% to 100%, but were generally around 70%. At the individual level, response rates were higher than 80% in most countries. Our analyses included only adolescents who indicated they had ever had sexual intercourse. Every participating country obtained approval to conduct the survey from the relevant ethics review board or regulatory body.

### Measures

#### *•Country‐level variable*

We used the Global Gender Gap Index for 2014 as a measure of country‐level gender equality.^39^ This index measures gender equality with respect to economic participation and opportunity (e.g., wage equality for men and women for similar work), educational attainment (e.g., ratio of females to males in primary, secondary and tertiary education), health and survival (e.g., female healthy life expectancy divided by male value) and political empowerment (e.g., ratio of female to male seats in parliament). Higher scores on the index indicate greater gender equality.

#### *•Individual‐level variables*

Respondents were asked if they had ever had sexual intercourse. In parentheses, other terms, such as “making love,” “having sex” and “going all the way,” were added to make sure respondents understood what was meant by “sexual intercourse”; these terms varied by country. Those who indicated that they had had intercourse were asked about their contraceptive use: “The last time you had sexual intercourse, did you or your partner use a condom?” and “The last time you had sexual intercourse, did you or your partner use birth control pills?” Response options were “yes,” “no” and “don't know.” On the basis of these two variables, a third variable was created, indicating the use of dual methods. Students who reported using both a condom and pills were classified as having used dual methods.

#### *•Covariates of contraceptive use*

All individual‐level variables relevant to adolescents’ contraceptive use that were available in the international HBSC data set were included in the analyses.

Age was included as a continuous variable. The HBSC Family Affluence Scale^40^, ^41^ was used as a proxy for socioeconomic status. The 2013–2014 survey used a six‐item assessment of common material assets and activities of an adolescent's family (e.g., “Does your family own a car, van or truck?” with response options of “no,” “one” and “two or more”; and “How many times did you and your family travel out of [country/region name] for a holiday/vacation last year?” with options of “not at all,” “once,” “twice” and “more than twice”). Responses were scored and summed. In this analysis, adolescents’ socioeconomic position was calculated by comparing the individual's summary score from the affluence scale with all other summary scores in the respective country or region. In so doing, we identified groups of young people in the lowest 20% (low affluence), middle 60% (medium affluence) and highest 20% (high affluence) in each country and region.^4^

Living arrangement was specified using four categories: with both biological parents, with a step family, with a single parent or in another situation. Classmate support was measured as the mean score on three items: “Students in my class(es) enjoy being together,” “Most of the students in my class(es) are kind and helpful” and “Other students accept me as I am.” Participants indicated to what extent they agreed with the items using five‐point Likert scales (Cronbach's alpha, 0.76). Possible scores ranged from 0 to 4; higher scores reflect higher perceived classmate support.

Substance use was measured with three items: the frequency of alcohol use in the last 30 days (on a seven‐point Likert scale ranging from “never” to “30 days”), frequency of current tobacco use (on a four‐point Likert scale ranging from “I do not smoke” to “every day”) and frequency of getting drunk in the last 30 days (on a five‐point Likert scale ranging from “never” to “more than 10 times”).

The measure of easy parent‐child communication was based on separate questions asking about respondents’ perceptions of communication with their mother and father about things that really bothered them (rated on a four‐point Likert scale ranging from “very easy” to “very difficult”). Each item was dichotomized (easy vs. difficult), and the more positive rating was used.

### Analysis

In total, 12,907 respondents in our sample indicated that they had had sexual intercourse. We excluded students who did not know if they had used contraceptives at last intercourse^i^ and those who had missing data on any analysis covariates. The percentages of missing data were low overall but varied across individual‐level variables: age (2%), family affluence (7%), living arrangement (3%), classmate support (1%), alcohol use (4%), current smoking (2%), drunkenness (4%), parent‐child communication (4%), condom use (5%) and pill use (11%). We compared the sampled students’ distribution by age, family affluence and gender with that of students who were excluded because of missing data. The mean age and the proportions of students with low, middle or high family affluence were very similar between both groups. However, the proportion who were male was higher in the excluded group than in the sample (69% vs. 50%). This suggests that among sexually experienced students, young men were more likely than young women to not answer questions about contraceptive use, risk behavior and family characteristics. The final sample comprised 4,071 females and 4,110 males.

Analyses were conducted using MLwiN 2.36. First, we examined the individual‐level sample characteristics. Second, we made descriptive comparisons of the prevalence of the contraceptive methods used and the levels of gender equality across countries. The third step entailed assessing whether the variation in contraceptive use across countries was statistically significant and, if so, testing whether it was related to countries’ level of gender equality. This was done by conducting multilevel multinomial logistic regression analyses stratified by gender, in which condom use, pill use and dual method use were compared with no contraceptive use. By using multilevel models rather than standard multinomial models, we accounted for the fact that respondents were clustered within countries; ignoring this clustering would have led to an overestimation of the associations between gender equality and contraceptive use.^40^

Our first model was an empty, or null, model, which assessed whether the variation in contraceptive use across countries was statistically significant (as indicated by the country intercept). The intraclass correlation coefficient indicated the amount of variance in contraceptive use that was due to differences across country contexts. The higher the coefficient, the more important the country level is in explaining contraceptive use. If the variation in use across countries is statistically significant and the intraclass correlation coefficient is considerable, variables can be added to the model. In model 1, we included individual‐level controls to assess associations between contraceptive use and these variables**.** In model 2, we added gender equality to assess its relationship with contraceptive use while controlling for the individual‐level variables.

Because most countries with high levels of gender equality are relatively wealthy,^22^ we wanted to identify any associations that reflected links with high national wealth rather than gender equality. Therefore, we performed sensitivity analyses to check the robustness of our findings after controlling for national‐level measures for wealth (gross national income per capita) and income inequality (the Gini coefficient).^42^ Furthermore, although all country samples were based on nationally representative data, some countries had relatively small samples of sexually experienced adolescents (fewer than 100 in Israel and Malta). As a second set of sensitivity analyses, we repeated the analyses while excluding these countries.

For conceptual reasons, analyses were conducted for males and females separately. To test whether the association between gender equality and adolescents’ contraceptive use differed significantly between young men and young women, an interaction analysis of “adolescent sex x gender equality” was performed on the combined data.

## RESULTS

### Sample Characteristics

Our sample was evenly split between males and females (Table 1), and the average age was 15.6 years (not shown). Most adolescents (58%) reported medium family affluence; 20% reported low and 22% high affluence. Sixty‐one percent lived with both biological parents, 21% with a single parent and 15% with a step family, and 3% had some other living arrangement. Sixty‐five percent of the students agreed or strongly agreed that they like being with their classmates, 61% that most students are kind and helpful and 73% that other students accept them as they are. Some 61% had used alcohol in the past 30 days, 22% smoked daily and 34% had been drunk at least once in the past month. Eighty percent of adolescents reported having easy communication with their parents about things that really bothered them. Overall, 19% had used no method at last intercourse; 49% had used a condom only, 11% the pill only and 21% dual methods.

**Table 1:** Percentage distribution of sexually experienced adolescents participating in the 2013–2014 Health Behaviour in School‐Aged Children (HBSC) study, by selected individual‐level variables

Variable	% (N = 8,181)
**Gender**	
Male	50.2
Female	49.8
**Family affluence**	
Low	19.9
Medium	57.7
High	22.4
**Living arrangement**	
With both biological parents	61.2
With step family	14.9
With single parent	20.6
Other	3.3
**Classmate support**	
“Students in my class(es) enjoy being together”	
Strongly agree	23.4
Agree	41.1
Neither agree nor disagree	25.2
Disagree	6.6
Strongly disagree	3.7
“Most of the students in my class(es) are kind and helpful”	
Strongly agree	19.7
Agree	41.6
Neither agree nor disagree	23.3
Disagree	10.2
Strongly disagree	5.2
“Other students accept me as I am”	
Strongly agree	32.5
Agree	40.3
Neither agree nor disagree	16.4
Disagree	6.2
Strongly disagree	4.6
**Frequency of alcohol use in past 30 days**	
Never	38.6
1–2 days	28.6
3–5 days	15.7
6–9 days	8.4
10–19 days	4.4
20–29 days	1.2
30 days	3.1
	
**Frequency of current tobacco use**	
I do not smoke	61.7
Less than once a week	8.8
At least once a week, but not every day	7.9
Every day	21.6
	
**Frequency of getting drunk in past 30 days**	
Never	65.9
Once	19.2
2–3 times	9.7
4–10 times	2.8
>10 times	2.4
	
**Easy parent‐child communication**	
No	20.3
Yes	79.7
	
**Contraceptive use at last sexual intercourse**	
Condom only	49.4
Pill only	11.2
Dual methods	20.8
None	18.7
	
Total	100.0

*Note*: Percentages may not total 100.0 because of rounding.

**Figure 1:**
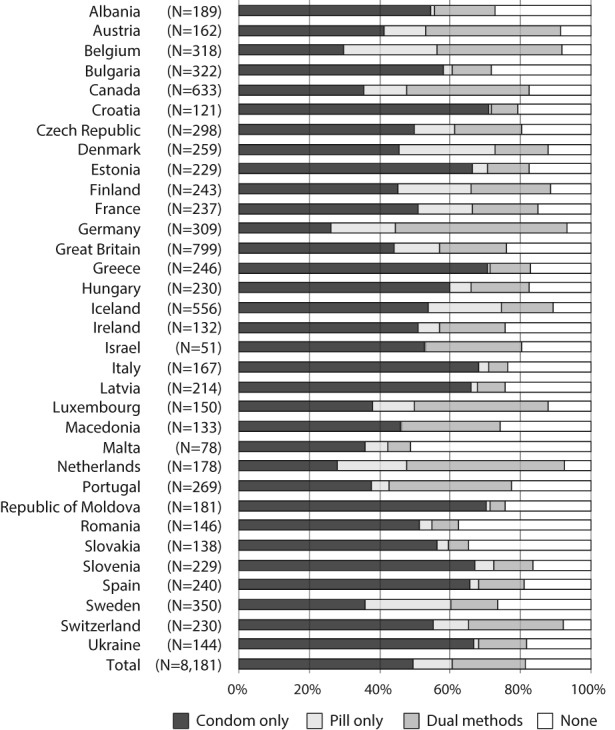
Percentage distribution of adolescents by contraceptive method used at last intercourse, according to country *Note*: There is significant (p < .001) variability across countries in the type of contraceptive used at last intercourse.

The contraceptive methods used at last intercourse varied significantly across countries (Figure 1). While condoms were the most commonly used method, Belgium, Germany, Scandinavian countries and the Netherlands had relatively high proportions of young people who used the pill (18–27%). Dual use was most prevalent in Austria, Belgium, Canada, Germany, Luxembourg, the Netherlands and Portugal (35–49%). Contraceptive nonuse was especially high in Malta (51%), Romania (38%) and Slovakia (35%).

**Figure 2:**
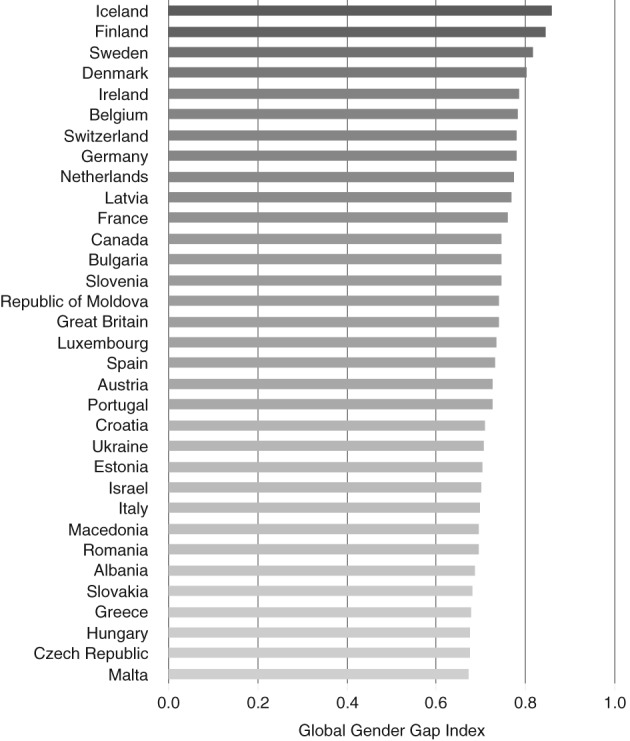
Global Gender Gap Index, by country, 2014 *Note*: The Global Gender Gap Index measures gender equality with respect to economic participation and opportunity, educational attainment, health and survival, and political empowerment.

Gender equality also varied significantly across countries (Figure 2). Global Gender Gap Index scores were highest in Nordic countries (e.g., Iceland, 0.86) and lowest in eastern and southern Europe (e.g., Malta, 0.67).

### Multilevel Findings

#### *•Young women*

In our null model, the country intercepts were all significant (p < .001), indicating that there was significant variation across countries in young women's contraceptive use (not shown). The intraclass correlation coefficients indicated that 7% of the variability in females’ condom use, 22% in their pill use and 19% in their use of dual methods were due to differences across country contexts. This suggests that variables at the national level play a considerable role in contraceptive use among young women.

In model 1, we included individual‐level covariates (Table 2). Young women living with a single parent were less likely than those living with both biological parents to have reported using a condom only at last intercourse (odds ratio, 0.8). Compared with young women who did not smoke, those who smoked at least once a week and those who smoked daily had reduced odds of condom use (0.6 and 0.7, respectively). Young women who reported being drunk more than 10 times in the past 30 days were less likely to report condom use than those who reported no drunkenness in that time period (0.3); the odds of condom use increased with level of classmate support (1.1 for each additional point on the scale). A single association was found for use of the pill only: The likelihood of such use increased with young women's age (1.4 for each additional year). As with condom use, the odds of dual method use rose with level of classmate support (1.2). In addition, dual use was more likely among young women who reported easy communication with their parents than among those who did not (1.3). Compared with young women who did not drink, those who drank alcohol daily were less likely to report dual use at last sex (0.3); young women who smoked were less likely than those who did not to have used dual methods (0.5–0.7, depending on the frequency of smoking). Finally, young women who reported 4–10 episodes of drunkenness in the last 30 days had reduced odds of dual method use compared with those who were never drunk in that time period (0.4).

**Table 2:** Odds ratios from multilevel multinomial logistic regression analyses assessing associations between young women's contraceptive use at last intercourse and selected individual‐level variables and gender equality

Variable	Model 1			Model 2		
	Condom only	Pill only	Dual methods	Condom only	Pill only	Dual methods
**Individual covariates**						
Age	1.0	1.4*	1.1	1.0	1.4**	1.2
Family affluence						
Low (ref)	1.0	1.0	1.0	1.0	1.0	1.0
Medium	1.0	0.9	1.1	0.9	0.7***	0.8
High	0.9	0.9	1.2	0.8*	0.6***	0.8
Living arrangement						
With both biological parents (ref)	1.0	1.0	1.0	1.0	1.0	1.0
With step family	0.9	1.0	1.0	0.7***	0.7***	0.7***
With single parent	0.8***	1.0	0.8	0.7***	0.6***	0.6***
Other	0.9	0.8	1.0	0.7*	0.3***	0.6**
Classmate support (range, 0–4†)	1.1***	1.1	1.2**	1.2***	1.2***	1.3***
Frequency of alcohol use in past 30 days						
Never (ref)	1.0	1.0	1.0	1.0	1.0	1.0
1–2 days	1.1	1.1	0.9	1.0	0.8	0.8*
3–5 days	1.0	1.2	1.0	1.1	1.3	1.1
6–9 days	0.9	1.0	1.0	0.9	0.9	0.9
10–19 days	1.1	1.3	1.0	1.1	1.5	1.1
20–29 days	0.7	0.8	0.7	0.7	1.0	0.7
30 days	0.8	0.5	0.3**	0.8	0.4	0.3**
Frequency of current tobacco use						
I do not smoke (ref)	1.0	1.0	1.0	1.0	1.0	1.0
Less than once a week	0.9	0.7	0.7*	0.7**	0.4***	0.5***
At least once a week, but not every day	0.6***	0.7	0.5***	0.5***	0.5***	0.4***
Every day	0.7***	0.9	0.6***	0.6***	0.6***	0.5***
Frequency of getting drunk in past 30 days						
Never (ref)	1.0	1.0	1.0	1.0	1.0	1.0
Once	1.0	1.0	1.0	1.0	1.0	1.0
2–3 times	0.9	0.9	0.9	0.7*	0.6**	0.6**
4–10 times	0.8	1.0	0.4*	0.6*	0.7	0.3**
>10 times	0.3 ***	0.6	0.8	0.3***	0.6	0.8
Easy parent‐child communication						
No (ref)	1.0	1.0	1.0	1.0	1.0	1.0
Yes	1.1	1.6	1.3*	1.3**	1.6***	1.8***
**Country predictor**						
Gender equality	na	na	na	2.1***	9.6***	5.6***
*Country intercept (standard error)*	*0.2 (0.1)*	*0.9 (0.3)*	*0.8 (0.2)*	*0.2 (0.1)*	*0.6 (0.2)*	*0.6 (0.2)*

* p < .05.

** p < .01.

*** p < .001.

† Higher scores indicate higher perceived classmate support. *Notes:* N = 4,071. ref = reference group. na = not applicable.

In model 2, we added the country‐level measure of gender equality. An increasing equality score was positively associated with condom use (odds ratio, 2.1), pill use (9.6) and dual method use (5.6) at last intercourse. Thus, sexually experienced young women living in relatively gender‐equal countries were more likely to report the use of all three contraceptive methods than their counterparts living in relatively gender‐unequal countries. For example, for every 0.1‐point increase on the gender equality scale, the odds of pill use increased by 860%. Thus, the odds of pill use among young women in Iceland, where the Global Gender Gap Index score is 0.86, were almost nine times those of young women in France, where the score is 0.76 (not shown). Most of the associations between contraceptive use and individual‐level variables became more pronounced after gender equality was added to the model.

#### *•Young men*

In the null model, the country intercepts were all significant (p < .001), demonstrating significant variation across countries in young men's contraceptive use (not shown). The intraclass correlation coefficients indicated that 8% of the variability in condom use, 21% in pill use and 13% in use of dual methods were due to differences across country contexts.

In model 1, young men's use of each contraceptive method at last intercourse was positively associated with increasing age (odds ratios, 1.3–2.2) and high family affluence (1.5–1.7—Table 3). In addition, use of condoms only was positively associated with classmate support (1.2); however, it was negatively associated with living without parents or step parents (0.7), daily alcohol use (0.6), daily smoking (0.8) and drunkenness at least 2–3 times in the past 30 days (0.4–0.7). Males’ dual method use was positively associated with medium family affluence (1.2), classmate support (1.2) and use of alcohol 10–19 days in the past month (1.5); it was negatively associated with daily smoking (0.7) and drunkenness 1–10 times over the past month (0.6–0.8). In model 2, increasing gender equality was positively related to the use of pills only (6.5) and dual methods (2.1) at last sexual intercourse. The association between gender equality and young men's condom use was marginally significant (1.4). After gender equality was added to the model, some of the associations at the individual level became more pronounced.

**Table 3:** Odds ratios from multilevel multinomial logistic regression analyses assessing the associations between young men's contraceptive use at last intercourse and selected individual‐level variables and gender equality

Variable	Model 1			Model 2		
	Condom only	Pill only	Dual methods	Condom only	Pill only	Dual methods
**Individual covariates**						
Age	1.5***	2.2***	1.3***	1.6***	2.3***	1.4***
Family affluence						
Low (ref)	1.0	1.0	1.0	1.0	1.0	1.0
Medium	1.2	1.3	1.2*	1.2*	1.4*	1.3*
High	1.5***	1.5*	1.7***	1.5***	1.7**	1.8***
Living arrangement						
With both biological parents (ref)	1.0	1.0	1.0	1.0	1.0	1.0
With step family	1.1	1.0	1.2	1.1	1.2	1.3*
With single parent	1.0	1.1	1.1	1.0	1.2	1.1
Other	0.7*	0.8	1.0	0.6**	0.5	0.9
Classmate support (range, 0–4‡)	1.2***	1.1	1.2***	1.2***	1.2*	1.2***
Frequency of alcohol use in past 30 days						
Never (ref)	1.0	1.0	1.0	1.0	1.0	1.0
1–2 days	1.1	1.0	1.1	1.1	0.9	1.0
3–5 days	1.0	0.8	0.8	1.0	0.8	0.8
6–9 days	1.0	0.8	1.0	1.0	0.8	1.0
10–19 days	1.3	1.5	1.5*	1.3	1.6	1.4
20–29 days	1.1	1.0	1.4	1.0	0.8	1.2
30 days	0.6*	1.3	1.0	0.6*	1.2	0.9
Frequency of current tobacco use						
I do not smoke (ref)	1.0	1.0	1.0	1.0	1.0	1.0
Less than once a week	0.9	1.2	1.1	0.9	1.1	1.1
At least once a week, but not every day	0.9	0.8	1.2	0.8	0.7	1.1
Every day	0.8**	0.7	0.7**	0.7***	0.6**	0.7***
Frequency of getting drunk in past 30 days						
Never (ref)	1.0	1.0	1.0	1.0	1.0	1.0
Once	0.9	1.4	0.8*	0.9	1.4*	0.8*
2–3 times	0.7**	1.3	0.7*	0.7**	1.1	0.6**
4–10 times	0.7*	1.6	0.6*	0.6*	1.5	0.6*
>10 times	0.4*	1.0	0.7	0.4***	1.0	0.6
Easy parent‐child communication						
No (ref)	1.0	1.0	1.0	1.0	1.0	1.0
Yes	1.1	1.4	1.2	1.1	1.4	1.2
**Country predictor**						
Gender equality	na	na	na	1.4†	6.5***	2.1**
*Country intercept (standard error)*	*0.3 (0.1)*	*0.8 (0.2)*	*0.5 (0.1)*	*0.3 (0.1)*	*0.3 (0.1)*	*0.4 (0.1)*

* p < .05.

** p < .01.

*** p < .001.

† p < .10.

‡ Higher scores indicate higher perceived classmate support. *Notes*: N = 4,110. ref = reference group. na = not applicable.

### Sensitivity Analyses

The interaction analysis of “adolescent sex x gender equality” with the combined data from young men and women confirmed that the associations between gender equality and contraceptive use were stronger for females than for males (odds ratio for condoms only, 1.3; for pills only, 1.9; for dual methods, 1.3; all p < .001).

Inclusion of national‐level measures for wealth and income inequality in the models did not change the results for pill use or dual method use, but did for condom use; the association lost significance for both genders. This suggests that condom use may be higher in gender‐equal societies because these countries are wealthier; young people may have better access to condoms in these countries. Excluding countries with relatively small samples (Israel and Malta) did not affect the main results or conclusions.

## DISCUSSION

The current study revealed that societal gender equality is positively associated with contraceptive use among adolescents, especially pill use. Even when national wealth and income inequality were taken into account, the association between gender equality and pill use remained significant. Thus, in countries with generally equitable wealth and income inequality levels, societal gender equality may ameliorate young women's risk for unwanted pregnancy. Such equality may be related to pill use in particular because the method is under the control of young women, and they may feel more empowered in relatively gender‐equal societies.

Our finding that the association between gender equality and pill use was stronger among young women than among young men may reflect the fact that young men may not always be aware of their partner's pill use. Even in relatively gender‐equal countries, such as Sweden, it is generally perceived that young women have a greater responsibility in avoiding pregnancy than do young men, who often put blind trust in women's use of hormonal contraceptives or emergency contraception.^43^ Because our data included only individual‐level, and not couple‐level, data, we could not assess whether young men in gender‐equal countries were more aware of their partners’ pill use than their counterparts in gender‐unequal countries. Future research should examine whether gender equality is linked to more openness in sexual communication between adolescent partners, which would result in greater awareness among young men regarding such contraceptive use.

Associations with the use of condoms only and dual methods were not as strong as those for use of pills only, and the association with condom use lost significance when national wealth and income inequality were considered. This finding may be explained in different ways. First, condoms are readily accessible through commercial establishments, and compared with the pill, there may be less stigma about obtaining them. Young women often have to visit a health care provider to get the pill; access difficulties present barriers that may be diminished in more gender‐egalitarian environments. Second, condom use is traditionally regarded as a male‐dominated method—one that men should take care of. This belief may prevail even in countries with high levels of gender equality, and may limit young women's confidence in getting actively involved in the decision‐making process regarding condom use. While young women can use the pill without having to negotiate with their partner, condom use is dependent on two decision makers.^44^ Although sexual communication between intimate partners tends to be better among adults in more gender‐egalitarian countries,^21^ talking about contraceptives remains a difficult and sensitive topic, especially among adolescents.^45^ Finally, an alternative explanation is that steady couples who use the pill may feel that condom use is not necessary. For these couples, not using a condom does not necessarily imply unsafe sex.

The observed association between macro‐level gender equality and adolescents’ contraceptive use is consistent with findings from empirical studies that examined adolescents’ contraceptive use and gender equality at an individual or micro level.^28^, ^29^, ^30^, ^31^ To our knowledge, ours is the first study to assess the relationship between macro‐level gender equality and adolescents’ contraceptive use. Future research should examine the extent to which the association between societal gender equality and contraceptive use among adolescents is mediated by potential mechanisms such as the availability of comprehensive sex education, a universal health care system, the accessibility of youth‐friendly health services and egalitarian gender norms.

Interestingly, we found that when controlling for gender equality, associations between individual‐level variables and contraceptive use became more pronounced, especially for young women. This suggests that suppressor effects are at play. Gender equality may make parent‐child communication about contraceptives easier (because of greater openness in more equal societies), and may lessen socioeconomic differences in contraceptive use (by removing physical and financial barriers). This explains why variables such as family affluence and parent‐child communication appear to be more strongly related to contraceptive use once gender equality is taken into account.

### Strengths and Limitations

This study has multiple strengths, such as the use of large, nationally representative data sets, the inclusion of many European countries and the use of multilevel analyses to quantitatively estimate associations between gender equality and adolescents’ contraceptive use. However, the study's findings should be interpreted with awareness of its limitations. First, our study was cross‐sectional, and therefore no causal inferences can be drawn on the basis of our results. Yet, given that gender equality was measured as a country‐level index reflecting objective measures in different societal domains (and was not based on the perceptions of adolescents in our sample), we avoided some potential problems of endogeneity (e.g., adolescents’ contraceptive use influencing self‐reported gender equality).

Second, contraceptive use was self‐reported and subject to reporting biases potentially related to cultural norms. If use was underreported in countries with lower gender equality, then our results may overestimate the association between equality and use.

Third, the use of a complete case analysis may result in selectivity biases. In particular, our comparison between the students in the final sample and those who were excluded because of missing data suggested that, compared with young women, sexually experienced young men may be less likely to report on their home environment, contraceptive use and risk behavior. As a result, we need to be cautious in drawing conclusions about differences between young men and young women in the relationship between gender equality and contraceptive use.

Fourth, we did not have concrete data on adolescents’ use of reliable methods other than the condom and the pill. In total, 16% of respondents reported having used a method other than these two. It is, however, unclear how many of these adolescents used reliable methods, because unreliable methods such as withdrawal also fall into the category “other methods.” From studies that have assessed IUD and implant use among 15‐year‐old females, the use of these methods seems to be very low. For instance, in a nationally representative study in the United States, 3% of 15–19‐year‐old women reported ever having used an IUD, and 3% an implant.^46^ According to a nationally representative Dutch study, 1% of 15‐year‐olds had used an IUD, and 1% an implant.^47^ Thus, we may have misclassified as nonusers a small number of young women who used an IUD or implant.

Finally, we were unable to control for some potentially important individual confounders, or mediators, such as knowledge of and attitudes toward contraceptive methods and the quality of communication with sexual partners, in relation to contraceptive use because of the limited availability of such measures within the HBSC data set. If future data sets become available that include such measures, replications of our analyses including these variables would be warranted.

## Conclusions

Having taken individual‐level and national economic variables into account, this study demonstrates that compared with their peers living in relatively gender‐unequal countries, sexually experienced adolescents living in relatively gender‐equal countries are more likely to report contraceptive use, especially pill use. These findings underscore that increasing safer sex practices among adolescents may require more than implementing individually oriented programs aimed solely at increasing contraceptive use. Rather, public health policy may need to adopt a society‐level perspective to address gender norms and equality. However, more research is needed to identify potential causal pathways and mechanisms through which gender equality and adolescents’ contraceptive use are linked.

## Footnotes

i The answer category “don't know” was chosen more often by adolescents in countries with low levels of gender equality than by those in countries with high levels (odds ratios, 0.01 for condom use and 0.07 for pill use; p < .001 for each). Also, young men were more likely than young women to be unsure about contraceptive use (2.5 for condom use and 9.8 for pill use; p < .001 for each).
